# A cytogenetic analysis in two species of Cassidinae (Coleoptera, Chrysomelidae)

**DOI:** 10.3897/CompCytogen.v13i3.36581

**Published:** 2019-09-03

**Authors:** Eduard Petitpierre

**Affiliations:** 1 Dept. Biologia, Universitat de les Illes Balears, 07122 Palma de Mallorca, Spain Universitat de les Illes Balears Palma de Mallorca Spain

**Keywords:** Coleoptera, Chrysomelidae, Cassidinae, *Cassida humeralis*, *Anacassis fuscata*, karyotypes

## Abstract

Two species of Cassidinae have been chromosomally analyzed, *Cassida humeralis* Kraatz, 1874 from France, with 2n = 18, 8 + Xy_p_ meioformula and *Anacassis fuscata* (Klug, 1829) from Uruguay, with 2n = 30, 14 + Xy meioformula. The karyotype of the former is composed of similar meta/submetacentric autosomes, a small X-chromosome and a tiny y-chromosome, as many other *Cassida* and tribe Cassidini species, whereas that of the latter has four pairs of acro/telocentric autosomes at least and the remaining meta/submetacentrics including the X-chromosome and a tiny y-chromosome, which points out to its probable apomorphic origin by centric fissions, as found in some other species of the tribe Mesomphaliini.

## Introduction

The leaf beetles of subfamily Cassidinae are a very large group with some 6,000 species distributed in 43 tribes (Chaboo 2007). Nearly 130 species have been chromosomally analysed mostly from the Palaearctic, Neotropical and Oriental regions (Petitpierre et al. 1988, Petitpierre et al. 1998; De Julio et al. 2010; Lopes et al. 2015; Lopes et al. 2017). Although the range of chromosome numbers is very large, from 2n = 12 to 2n (♂) = 51, roughly 40% of their species show 2n = 18 chromosomes (De Julio et al. 2010). Moreover, the sex-chromosome system in males is the “parachute type” Xy_p_, as found in nearly 95% of Cassidinae (De Julio et al. 2010) and in most beetles of the suborder Polyphaga (Smith and Virkki 1978). The present paper is a small contribution to the cytogenetics of Cassidinae and a brief discussion on its chromosomal evolution.

## Material and methods

Two species of Cassidinae, each from two individuals, have been chromosomally surveyed: *Cassida humeralis* Kraatz, 1874, from Revens (Gard, France) and *Anacassis fuscata* (Klug, 1829) from Sauce (Canelones, Uruguay). The male adult individuals were anaesthetized with ethyl acetate before dissecting their testes with insect pins and using then the methods of chromosome treatments reported by Petitpierre et al. (1998), for obtaining chromosome spreads which were conventionally stained with Giemsa. Finally, the slides were examined and photographed with a Zeiss Axioskop photomicroscope.

## Results

**Figures 1–4.**
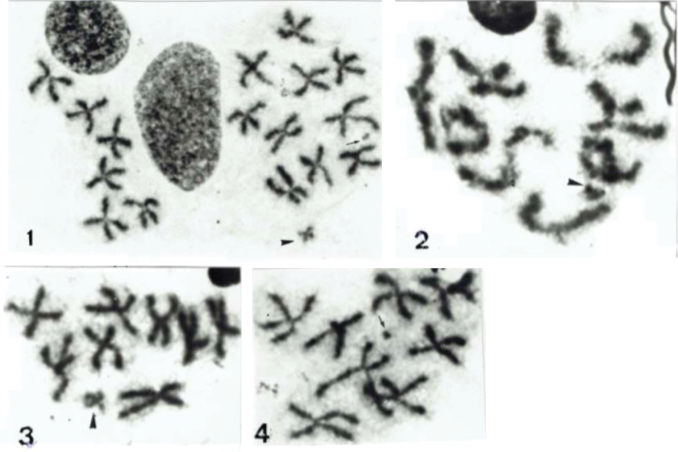
*Cassida humeralis*: **1** spermatogonial metaphase with 2n = 18 meta/submetacentric chromosomes, the small X-chromosome is arrowheaded and the tiny y-chromosome pointed by an arrow **2** meiotic diakinesis with a 8 + Xyp meioformula, the Xyp is arrowheaded **3** meiotic metaphase II of X-chromosome (arrowheaded) class with nine chromosomes **4** meiotic metaphase II of y-chromosome (arrowed) class with nine chromosomes.

*Cassida humeralis* provided spermatogonial metaphases with 2n = 18 chromosomes, all the autosomes showing similar sizes and metacentric shapes except one pair of submetacentrics, whereas the X sex-chromosome was a clearly smaller metacentric with regard to all autosomes and the y-chromosome a tiny element (Fig. 1). The meiotic metaphase I displayed an 8 + Xy_p_ formula, with rings of two chiasmata, cross and rod-shaped one-chiasma autosomal bivalents in variable numbers and the Xy_p_ sex-chromosome system (Fig. 2). The meiotic metaphases II showed as expected the two classes, with 8 + X and 8 + y haploid chromosome numbers, respectively (Figs 3, 4).

**Figures 5–8.**
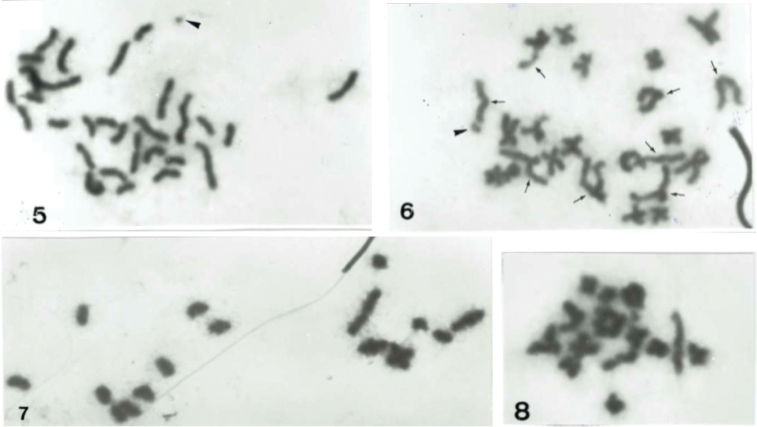
*Anacassis fuscata*. **5** spermatogonial metaphase with 2n = 30 chromosomes **6** spermatogonial metaphase showing at least four acro/telocentric autosome pairs indicated by arrows **7** meiotic metaphase I with a 14 + Xy meioformula **8** meiotic metaphase II of X-chromosome class with fifteen chromosomes.

*Anacassis fuscata* had spermatogonial metaphases with 2n = 30 chromosomes of mostly medium and small sizes (Fig. 5), at least four pairs of them acro/telocentrics and the remaining meta- or submetacentrics including the X-chromosome, and a tiny y-chromosome (Fig. 6). The meiotic metaphase I displayed a 14 + Xy meiformula (Fig. 7), and a metaphase II showed 14 + X haploid chromosomes (Fig. 8).

## Discussion and conclusions

The karyotype of *Cassida humeralis* has 2n = 18 chromosomes as in 23 (69.7%) of the 33 cytogenetically known species of the genus *Cassida*, and in 40 (65.6%) among the total of 61 checked species of the tribe Cassidini, including species of further fourteen genera (Petitpierre et al. 1998; De Julio et al. 2010; Lopes et al. 2016, 2017). The prevalent metacentric shape of most autosomes in *C. humeralis* is also in agreement with those found in five other species of the same genus and in other genera of Cassidini tribe as well as a small metacentric X-chromosome and a tiny Y-chromosome (Petitpierre 1977; Petitpierre et al. 1998; De Julio et al. 2010). De Julio et al. (2010) assumed that a meioformula of 9 + Xy_p_ (2n = 20), the probable most ancestral for coleopterans of the suborder Polyphaga (Smith and Virkki 1978; Dutrillaux and Dutrillaux 2009), might also be the ancestral for the subfamily Cassidinae, but although this is present in three species of *Cassida* and in three further ones of different genera in the tribe Cassidini too (De Julio et al. 2010), it seems clear that it could not be the basal one. Moreover, the range of diploid numbers in the tribe Cassidini is quite large from 2n = 16 in *Glyphocassis trilineata* (Hope, 1831) to 2n = 42 in *Agroiconota inedita* (Boheman, 1855), but all out of one checked species in this tribe show the Xy_p_ sex chromosome system in males, although a few of them are polymorphic for an additional y_p_ chromosome (De Julio et al. 2010).

The high chromosome number, 2n = 30, of *Anacassis fuscata*, is in agreement with others found in the Neotropical tribe Mesomphaliini (= Stolaini), whose range in numbers goes from 2n = 22 to 2n(♂) = 51, in 24 checked species of six genera, where ten species of them had diploid numbers ≥ 30 chromosomes (De Julio et al. 2010; Lopes et al. 2016, 2017). *Anacassis fuscata* shows at least four acro/telocentric autosome pairs which points out to their possible origin from meta- or submetacentric autosomes by centric fissions, as it is found in other species of the tribe Mesomphaliini (De Julio et al. 2010), and a tiny y-chromosome, but the X-chromosome was not distinguished. Nevertheless, *Anacassis fuscata* displays a simple sex-chromosome system Xy (probably Xy_p_) in males, as occurs in species of other genera of this tribe, namely of *Chelymorpha* Chevrolat, 1837 , *Cyrtonota* Chevrolat, 1837, *Mesomphalia* Hope, 1839, *Paraselenis* Spaeth, 1913 and *Stolas* Billberg, 1820 (De Julio et al. 2010; Lopes et al. 2016, 2017), contrary to the highly complex sex-chromosome systems described in most species and chromosomal races of *Botanochara*, which are undoubtedly derived from the former simple one Xy_p_ by chromosomal rearrangements (De Julio et al. 2010; Lopes et al. 2017). Thus, the tribe Mesomphaliini is strikingly apomorphous from cytogenetic grounds, both due to the high diploid chromosome numbers and the highly complex sex-chromosome systems of a fair number of its species.

Eventually, the possible most ancestral karyotype for the whole Cassidinae s. lat. subfamily, that is including the ancient subfamilies of Cassidinae s. str. (tortoise beetles) and Hispinae (leaf-mining beetles), may be that of 2n = 18 (Xy_p_) chromosomes, because it is prevalent in two of the three tribes with at least five or more examined species of Cassidinae s. str., and in three of the six so far checked tribes belonging to the ancient subfamily Hispinae. However, in order to secure the basal karyotype of Cassidinae s. lat., many more species, most of all in this latter group of the ancient subfamily Hispinae, would be necessary to confirm this point of view.
